# Differential Expression of Core Metabolic Functions in *Candidatus Altiarchaeum* Inhabiting Distinct Subsurface Ecosystems

**DOI:** 10.1111/1758-2229.70096

**Published:** 2025-06-03

**Authors:** Sarah P. Esser, Victoria Turzynski, Julia Plewka, Julia Nuy, Carrie J. Moore, Indra Banas, André R. Soares, Janey Lee, Tanja Woyke, Alexander J. Probst

**Affiliations:** ^1^ Environmental Metagenomics Research Centre One Health Ruhr of the University Alliance Ruhr, Faculty of Chemistry, University Duisburg‐Essen Essen Germany; ^2^ Centre of Water and Environmental Research (ZWU) University of Duisburg‐Essen, Universitätsstraße 5 Essen Germany; ^3^ DOE Joint Genome Institute, Lawrence Berkeley National Laboratory Berkeley California USA; ^4^ Department of Life and Environmental Sciences University of California Merced Merced California USA; ^5^ Center for Medical Biotechnology (ZMB) University of Duisburg‐Essen Essen Germany

**Keywords:** archaea, metatranscriptomics, subsurface ecosystems

## Abstract

*Candidatus Altiarchaea* are widespread across aquatic subsurface ecosystems and possess a highly conserved core genome, yet adaptations of this core genome to different biotic and abiotic factors based on gene expression remain unknown. Here, we investigated the metatranscriptome of two *Ca. Altiarchaeum* populations that thrive in two substantially different subsurface ecosystems. In Crystal Geyser, a high‐CO_2_ groundwater system in the USA, *Ca. Altiarchaeum* crystalense co‐occurs with the symbiont *Ca. Huberiarchaeum* crystalense, while in the Muehlbacher sulfidic spring in Germany, an artesian spring high in sulfide concentration, *Ca. A*. *hamiconexum* is heavily infected with viruses. We here mapped metatranscriptome reads against their genomes to analyse the in situ expression profile of their core genomes. Out of 537 shared gene clusters, 331 were functionally annotated and 130 differed significantly in expression between the two sites. Main differences were related to genes involved in cell defence like CRISPR‐Cas, virus defence, replication, transcription and energy and carbon metabolism. Our results demonstrate that altiarchaeal populations in the subsurface are likely adapted to their environment while influenced by other biological entities that tamper with their core metabolism. We consequently posit that viruses and symbiotic interactions can be major energy sinks for organisms in the deep biosphere.

## Graphical abstract

Major differences between *Candidatus Altiarchaeum* species living in two distinct subsurface ecosystems as determined via metatranscriptomics. Genes overly expressed in different *Ca*. *Altiarchaea* in Muehlbacher sufidic spring (magenta; MSI; Regensburg, Germany) and Crystal Geyser (turquise; CG; Utah, USA). *Ca. Altiarchaeum hamiconexum* communities in MSI overexpressed genes encoding for phage shock proteins, signal peptide peptidases and FtsZ compared to *Ca. Altiarchaeum* crystalense in CG. In CG, the latter has an episymbiont (Probst et al. 2018 *Nature Microbiology* 3: 328–336; Esser et al. 2023 *Nature Microbiology* 8: 1619–1633) and exhibited a comparatively higher expression of genes encoding for CRISPR‐Cas, polysaccharide biosynthesis proteins and modification methylase MboII.

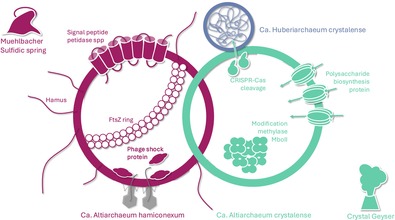

## 1 Introduction

**FIGURE 1:**
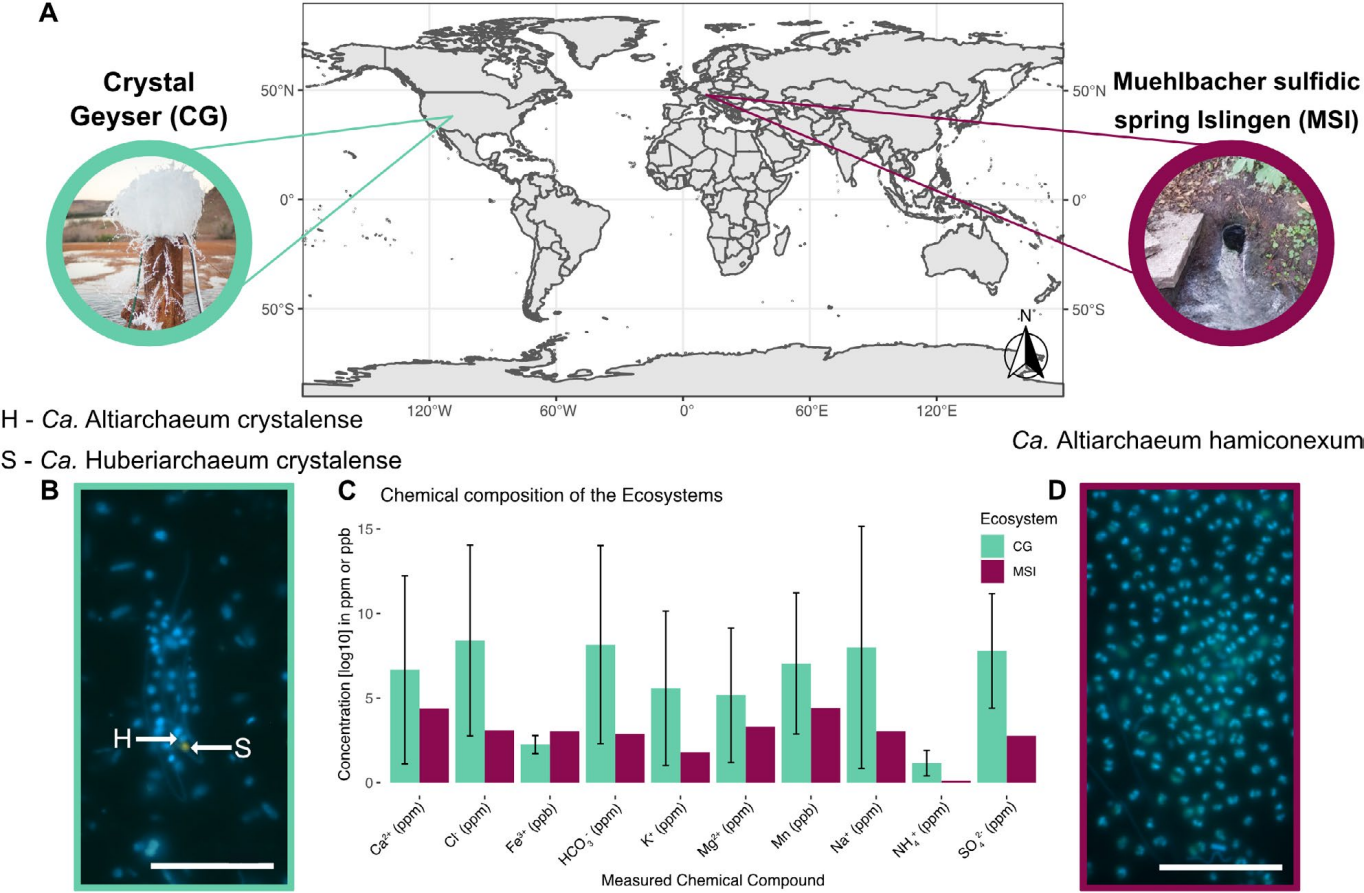
(A) World map showing the two sampling sites, crystal geyser (CG, aquamarine) and muehlbacher sulfidic spring (MSI, magenta). (C) Differences in the chemical composition of MSI (*n* = 1) (Rudolph et al. 2004), and CG (*n* = 3) (Probst et al. 2018) are shown for nutrients and ions measured in both ecosystems. The bars for CG show the average ppm/ppb of three samples (CG05; CG08; CG16) (Probst et al. 2018). (B and D) Fluorescence in situ hybridization (FISH) images show the host *Ca. Altiarchaeum* (SMARCH714 labelled with Atto448 (Rudolph et al. 2004)) in green (H) and the symbiont *Ca. Huberiarchaeum* (HUB1206 with Cy3 (Schwank et al. 2019)) in orange (S). Shown chemical compositions from CG were sampled in the minor eruption phase of the eruption cycle, where *Ca. Altiarchaea* is the dominant organism. Please note that the symbiont was not detected in MSI. Scale bar 10 μm.

The aquatic deep subsurface houses some of Earth's most diverse and complex ecosystems, varying in chemical, physical and geological parameters. As a result, they serve as ecological niches for differently adapted microorganisms. DPANN (Diapherotrites, Parvarchaeota, Aenigmarchaeota, Nanohaloarchaeota and Nanoarchaeota) archaea (Rinke et al. 2013) are alongside other Archaea and Bacteria found in various aquatic deep subsurface ecosystems such as terrestrial geysers, aquifers and boreholes (Huber et al. 2002; Castelle et al. 2015; Ortiz‐Alvarez and Casamayor 2016; Momper et al. 2017; Dombrowski et al. 2019). To cope with their limited metabolic capacity, DPANN archaea are known to be able to live in symbiosis with other archaea, with the best‐studied example of *Ignicoccus hospitalis* and *Nanoarchaeota equitans* (Huber et al. 2002; Jahn et al. 2004, 2008; Paper et al. 2007), but also recently described DPANN‐host associations from the branch of Micrarchaeota (Sakai et al. 2022). One exception to this rule is *Ca. Altiarchaeum*, a geographically widespread genus of Organisms that live freely as carbon‐fixing organisms in the deep subsurface. Recent investigations of *Ca. Altiarchaea* have shown site‐specific genomic adaptations likely resulting from horizontal gene transfer, yet these organisms harbour a highly conserved core genome that follows a strict biogeographic pattern (Bornemann et al. 2022). Originally discovered in the Sippenauer Moor associated with sulfur‐oxidising bacteria (Rudolph et al. 2001), *Ca. Altiarchaeum* dominates and lives freely in groundwater aquifers, e.g., Geyser Andernach (Bornemann et al. 2022) in Germany. However, the archaeon was also shown to be the host of *Ca. Huberiarchaeum* in very specific aquatic environments, i.e., Crystal Geyser (CG) in the US (Probst et al. 2018; Schwank et al. 2019; Esser et al. 2023) and the Horonobe Underground Research Laboratory in Japan (Hernsdorf et al. 2017). Here, we focus on metatranscriptomic expression profiles of *Ca. Altiarchaeum* with its episymbiont *Ca. Huberiarchaeum* present in Crystal Geyser (CG) (Probst et al. 2018; Schwank et al. 2019; Esser et al. 2023) compared to Muehlbacher sulfidic spring (MSI), where the symbiont is absent (Probst et al. 2014) (Figures 1 and S2) and *Ca. Altiarchaea* is heavily targeted by viruses (Rahlff et al. 2021; Turzynski et al. 2023; Banas et al. 2023). In general, metatranscriptomic datasets from deep subsurface inhabiting microorganisms are rare (Vuillemin et al. 2020; Murakami et al. 2012; Lopez‐Fernandez et al. 2018; Seyler et al. 2021) and the here presented datasets expand the knowledge of the active metabolic capacity within the deep subsurface. We hypothesised that the differences in the expression profile of *Ca. Altiarchaea* population genomes were not only influenced by the differing chemical composition of the two ecosystems but also by the presence of the episymbiont and/or viruses.

## 2 Results and Discussion

The two sampling sites, CG and MSI, are located in Utah, USA (N 38 56′ 18.125″; W 110 8′ 7.389″) and in Regensburg, Germany (N 48 59′ 8.999″; O 12 7′ 38.459″), respectively, and also differ in the chemical composition of bio‐processable ions and other molecules in their groundwater (Rudolph et al. 2001; Probst et al. 2018) (summarised in Figure 1A,C; Table 1). Particularly, the chemical composition of CG varies throughout the phases of eruption (Probst et al. 2018), whereby *Ca. Altiarchaea* is dominant in the minor eruption phase fed by the deepest intersected aquifer (Rudolph et al. 2001; Probst et al. 2018). When comparing the ion composition of CG's minor eruption phase to MSI, the latter sampling site appears to be rather limited in ion and nutrient availability (Figure 1C). MSI, as a sulfidic spring with high levels of sulfide (16 mg/L) (Rudolph et al. 2004), has a three‐fold logarithmic lower sulfate concentration than CG (average of 2209 mg/L from three sampling timepoints in the minor eruption phase) (Probst et al. 2018) (Table 1). Interestingly, nitrate, sodium, and potassium have also a lower abundance in MSI compared to CG, which might influence the necessity to form biofilms for nutrient retention and filtration from groundwater (Table 1). This is in agreement with the finding that *Ca. Altiarchaea* are predominantly present as single cells in CG (Probst et al. 2018) (Figures 1 and S1) and almost solely found as biofilms in MSI (Figures 1 and S2), where the cells interconnect with *hami* (Probst et al. 2014; Rahlff et al. 2021; Probst and Moissl‐Eichinger 2015). Irrespective of the mentioned physiological differences and site‐specific horizontal diversification of *Ca. Altiarchaea*, their core genome is highly conserved across many deep subsurface sites (Bornemann et al. 2022) rendering it the ideal genus for studying differential gene expression with respect to different environmental conditions. Besides a single report on the chemical composition of the groundwater at MSI (Rudolph et al. 2004), pH (7) and temperature (10.5°C) values were stable during multiple samplings over the past years and also in November 2022 when the samples for the metatranscriptome were leveraged.

**TABLE 1:** Compilation of nutrients, ions and bio‐accessible compounds measured in both ecosystems crystal geyser (Probst et al. 2018) (CG) and muehlbacher sulfidic spring islingen (Rudolph et al. 2004) (MSI).

Sample information			Nutrients, ions and bio‐accessible compounds										
Sample		Year	SO_4_^2−^ (ppm)	NH_4_^+^ (ppm)	Na^+^ (ppm)	Cl^−^ (ppm)	Mg^2+^ (ppm)	K^+^ (ppm)	HCO_3_^2−^ (ppm)	Ca^2+^ (ppm)	Fe^3+^ (ppb)	Mn (ppb)	Citation
CG	CG05	2015	2395	4.95	2383	4241	150	213	3695	660	10.16	1213	(Probst et al. 2018)
	CG08	2015	2389	0.82	2098	4388	138	204	3667	616	9.16	1083	
	CG16	2015	2443	3.78	4446	4780	236	374	3070	1087	9.13	1152	
MSI		2004	16	0.13	21	22	27	6.1	18	79	21	81	(Rudolph et al. 2004)

*Note:* Previously published values of MSI have been converted from mg/L into ppm or ppb, respectively.

### 2.1 Substantially Different Gene Expression Profiles in Two Separate Subsurface Ecosystems

**FIGURE 2:**
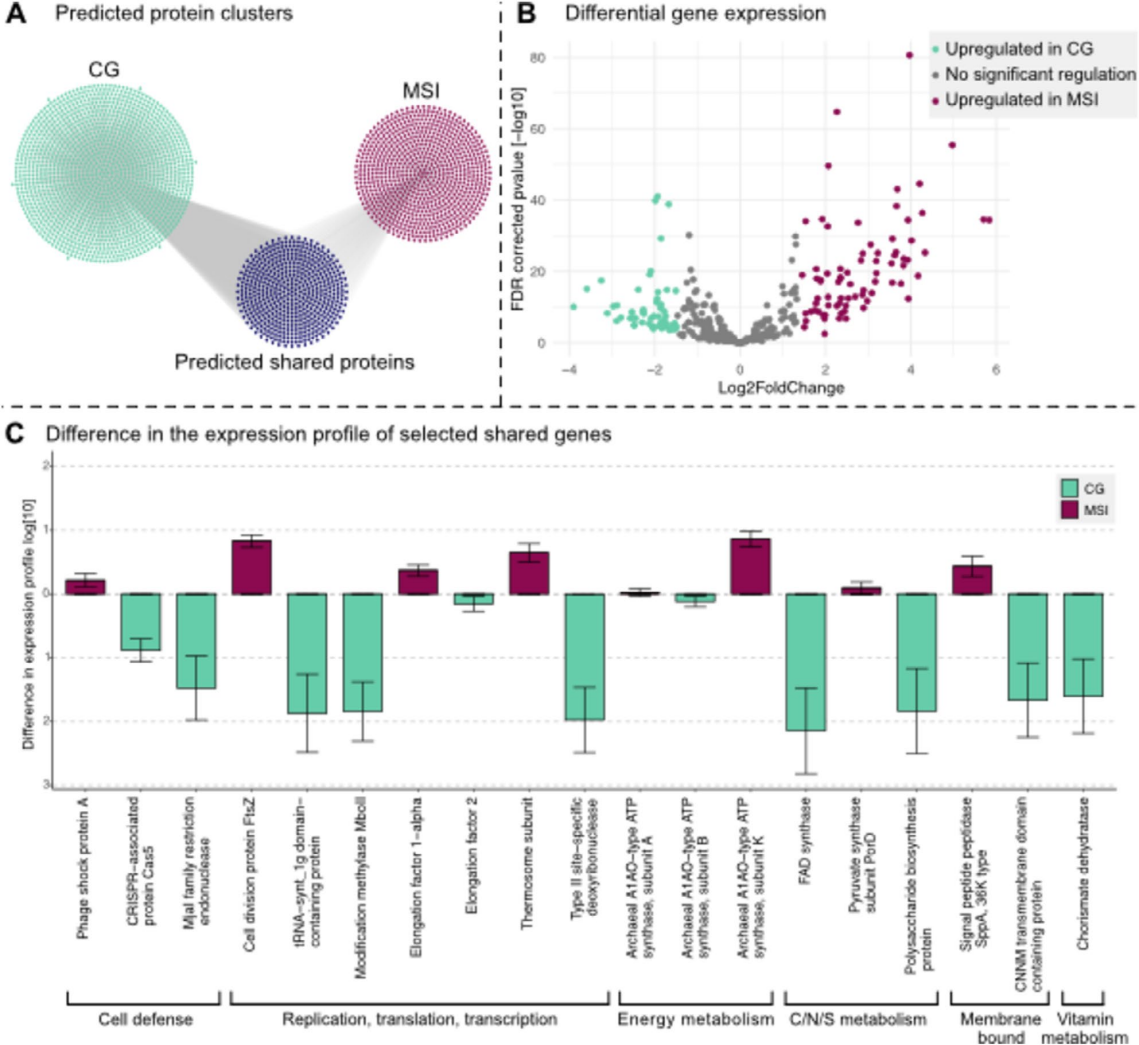
Gene clusters, differential gene expression and differences in gene expression profile of two *Ca. Altiarchaea* populations. (A) Gene clusters (80% AA similarity) of CG (*n* = 1447) and MSI (*n* = 824), as well as shared protein clusters (*n* = 537). (B) Differential gene expression of MSI compared to CG. The count data was normalised based on the coverage of 10 ribosomal proteins (see methods) and then evaluated with DESeq2 (Love et al. 2014; Liu et al. 2021) in R studio (Core Team 2013). (C) Difference graph of the expression profile for 19 shared gene clusters selected based on annotation, difference, and relationship to physiology/ecology (the values represent the difference in the expression of the gene clusters). All values, including mean expression rate and standard deviations, are listed in Table S3.

Predicted genes of previously published genomes from CG and MSI (14 genomes and one genome, respectively; Table S2) were clustered at 80% nucleotide similarity. Of the 537 shared gene clusters (Figure 2A), 430 were assigned a functional annotation. The 107 remaining gene clusters were either not annotated (no hit in FunTaxDB 1.2, which is based on UniRef100 release 2023_02 (Suzek et al. 2007; Suzek et al. 2015)) or annotated as uncharacterized proteins. From the 430 annotated gene clusters, 94 were within the first or last 200‐bp of the respective scaffold, causing irregularities in transcriptome mapping (underestimation of coverage). These genes were also excluded from downstream statistical analysis. The remaining 336 gene clusters were sorted according to their functional annotation, whereby the overall differential expression for most gene clusters in CG (~90.8%, *n* = 305) was higher than in MSI (~9.2%, *n* = 31).

Based on DESeq2 calculated Log2FoldChange (below −1.47 and above 1.44; FDR corrected *p* values < 0.05), the overall regulation of genes revealed a significant upregulation of 76 genes in MSI compared to 54 genes in CG. In general, the Log2FoldChange calculated based on DESeq2 approaches is generated by an estimation of gene‐wise dispersion and fits a respective model. This model is based on the baseline data, in this case, MSI transcriptional expression data. Genes overexpressed in CG code for proteins that included Modification Methylase MboII, a Type II site‐specific deoxyribonuclease, a FAD synthase, and an FAD synthase (Figure 2C). By contrast, MSI upregulated genes included those for ribosomal proteins (e.g., S11, S28e, L35Ae), a phage shock protein, a cell division protein (FtsZ) and a Thermosome subunit (Figure 2C).

### 2.2 Gene Expression Profiles Correlate With Physiological Characteristics

Focusing on the difference in the expression profile of shared gene clusters (Figures 2C and S3–S10) it was evident that the increase in expression of any given significantly differently expressed gene was substantially greater in CG than in MSI (Figures S11 and S12). Particularly, some genes related to replication (e.g., DNA polymerases, Tables S3 and S4) and nutrient metabolism have up to two‐fold higher expression in CG than in MSI (Figures S5 and S8), suggesting that *Ca. A*. crystalense is more active and replicating than *Ca. A*. *hamiconexum* when sampling the respective ecosystem. However, the expression of FtsZ, a protein involved in forming the septum of a dividing *Ca. A*. *hamiconexum* (Probst et al. 2014; Rahlff et al. 2021; Probst and Moissl‐Eichinger 2015) cell seems upregulated in MSI (Figure 2C). The accumulation of this protein, which usually has similar concentrations in the cell irrespective of cell division, can be used as an indicator of a starting cell division process, as it localises as a mid‐cell ring early in the division process shown for *E. coli* (Den Blaauwen et al. 1999). In addition, genes for the elongation factor 1 alpha, which is included in the aminoacyl tRNA incorporation in Archaea and Eukaryotes (Xu et al. 2022), and the thermosome subunit, which represents the chaperonin family in archaea and is accordingly involved in protein folding (Phipps et al. 1991, 1993), are up to two‐fold higher expressed in MSI as compared to CG. This can also be an indicator that cell division in MSI is upregulated. Therefore, the upregulation of ftsZ gene expression suggests a higher replication rate of *Ca. Altiarchaeum hamiconexum* in MSI and supports the visible diploidy of the cells in previously published (Probst et al. 2014; Henneberger et al. 2006) and here shown fluorescence in situ hybridisation (FISH) images (Figures 1, S1 and S2). Comparing the function of the cellular replication genes upregulated in CG (i.e., genes encoding DNA polymerases) versus MSI (i.e., genes encoding for the cell division process), it is indicated that *Ca. A*. crystalense appears to heavily replicate the genome but somehow does not proceed with the cell division cycle as corresponding cells in MSI. Previous studies on archaeal cell division showed that the depletion of the FtsZ proteins in *Haloferax volcanii* inhibits cell division, while DNA replication is still ongoing (Liao et al. 2021). We propose that although DNA synthesis is very prominent in Altiarchaea from CG, their final cell division seems hampered, likely due to the presence of the symbiont *Ca. H*. crystalense or many different viruses in CG that show infection histories with this organism (Esser et al. 2023).

### 2.3 Differential Expression of Microbial Defence Mechanism Relates to Types of Infection

*Ca. A*. *hamiconexum* has been described to be infected by at least two different viruses, one of which is a lytic virus (Rahlff et al. 2021; Turzynski et al. 2023). In agreement with these findings, the differential expression analysis revealed a significant increase in the gene encoding phage shock proteins, which are a stress response to membrane penetration by invading mobile genetic elements (MGEs) (Brissette et al. 1990), in MSI compared to CG. By contrast, CRISPR Cas 5, which is a protein involved in the cascade building for the splicing mechanisms in CRISPR type I systems (reviewed by Hille and Carpentier, 2016) (Hille and Charpentier 2016) is upregulated in *Ca. A*. crystalense (Figure S2C), which shows an expansive spacer variety over 6 years not only against MGEs but also against its episymbiont (Esser et al. 2023). Consequently, we identified a specific adaptation of defence mechanisms against lytic viruses and the episymbiont, respectively.

Beyond upregulation of the CRISPR system, the interaction of the episymbiont might also be responsible for the upregulation of other metabolic functions in *Ca. A*. crystalense. For example, multiple genes encoding proteins involved in energy metabolism, such as the quinolinate synthase, FO synthase subunit 2 and the FAD synthase (Figures 2C, S5 and S6) were significantly enriched in the CG transcriptome. Increased energy demands might stem from the highly active CRISPR Cas system, which acquired hundreds of thousands of different spacers in the *Ca. Altiarchaeum* population at CG (Esser et al. 2023). In addition, polysaccharide biosynthesis genes were found to be upregulated in CG, although we only seldom found biofilms of *Ca. Altiarchaea* in CG compared to MSI (Figure 1) (Probst et al. 2018, 2014). This upregulation could either be related to the scavenging nature of the episymbiont (Schwank et al. 2019) or indicate an intrinsic tendency of the *Ca. A*. crystalense to form biofilms without success, potentially due to the turbulent geyser system (Probst et al. 2018).

In summary, we found that gene expression of two *Ca. Altiarchaea* populations, originating from distinct geological settings, is influenced by environmental factors, such as high pressure and different geochemical conditions, as well as biological interactions. While the upregulated genes in the microbial population in MSI appear reflective of viral attacks, the episymbiont in CG and the turbulence of the geyser system seem to upregulate the CRISPR system, the energy metabolism and the biofilm formation. Leveraging metatranscriptomes of low‐biomass deep subsurface ecosystems, this study contributes to the existing DNA sequencing‐based body of literature on the deep biosphere in general and on *Ca. Altiarchaeum* in particular.

#### 2.3.1 Experimental Procedures

##### 2.3.1.1 RNA Extraction and Sequencing

For RNA extraction from *Ca. Altiarchaeota* biofilms in MSI, the biofilm flocks were harvested in November 2021 as previously described (Probst et al. 2013) and directly frozen at –80°C until further processing in the lab. RNA was extracted with the RNeasy PowerBiofilm RNA extraction kit (Qiagen, Germany) according to the manufacturer's instructions. The extracted RNA was sequenced at the LCSB (Luxembourg) with 150 bp paired‐end Illumina technology. Prior to sequencing, the rRNA was depleted using Zymo‐Seq RiboFree Total RNA Library Kit (R3003) according to the manufacturer's protocol. The library amplification was performed with 12 PCR cycles.

Sequencing data from Crystal Geyser was retrieved from a previous study (Esser et al. 2023) (minor eruption phase, samples CG05, CG08 and CG16), specifically using datasets from samples where *Ca. Altiarchaeum* crystalense is the most abundant organism. In brief, the erupted water from CG was sequentially filtered on PTFE filters, which were frozen on site on dry ice (Probst et al. 2018). The RNA extraction was performed with an adjusted protocol of the Qiagen DNeasy PowerMax Soil kit (Qiagen, Germantown, MD) (Esser et al. 2023). All accession numbers are listed in Table S1.

##### 2.3.1.2 Coverage‐Based Normalisation of Metatranscriptomes

After quality filtering with bbduk (https://github.com/BioInfoTools/BBMap/blob/master/sh/bbduk.sh) and sickle (Joshi and Fass 2011), the metatranscriptomics reads were normalised by mapping (Langmead and Salzberg 2012) reads against representative genomes of *Ca. Altiarchaeum hamiconexum* (MSI) and *Ca. A*. crystalense (CG) (see Table S1 for accession numbers). The mean coverage of 10 house‐keeping genes [30S ribosomal proteins: S4, S5, S7, S8e, S9, S10, S11, S12, S13, S15] was used to calculate the normalisation factor of each metatranscriptome sample (per ecosystem *n* = 3) (Table S2). Prior to choosing these genes for normalisation, the position of the gene on the scaffold (not within the first/last 200 bp of the scaffold) and a stable coverage distribution across the gene were taken into consideration. Despite the presence of complex microbial communities within the described ecosystems, the metatranscriptomic datasets mapped sufficiently to *Ca. Altiarchaeum*, as it is the dominant organism within the samples analysed herein.

##### 2.3.1.3 Clustering of Genes and Calculation of Expression Profile

Genes of the abovementioned *Ca. A. hamiconexum* and *Ca. A*. crystalense genomes were predicted with prodigal (Hyatt et al. 2010), and consecutively clustered with cdhit (Li and Godzik 2006; Fu et al. 2012) at 80% amino acid similarity (−c 0.8). The genes from the two ecosystems sharing a cluster were annotated with the FunTaxDB (Bornemann et al. 2023) (version 1.2) database. All genes that either had no or an unclassified annotation and/or which started or ended within the first or last 200 bps of the scaffolds were discarded within the expression profile. Removing genes starting or ending within the first or last 200 bps of a scaffold was chosen to avoid mapping distortions resulting from inaccurate mapping at these scaffold regions.

The abovementioned normalisation factors were used to determine the coverage differences introduced by the varying extraction and sequencing methods and the mean coverage with standard deviation of the shared gene clusters was calculated. The visualisation was performed with ggplot2 (Wickham 2016) in R studio (Core Team 2013; Posit team 2022) (version 2023.03.0 + 386). The data for evaluating the count data within the RNA‐seq data was performed with DESeq2 implemented in R (Love et al. 2014). The threshold values of up‐ and downregulation of differential gene expression were calculated to the base expression of MSI according to Quackenbush 2002 (Quackenbush 2002).

##### 2.3.1.4 Fluorescence In Situ Hybridisation (FISH)

*Ca. A*. hamiconexum biofilm flocks were taken from MSI. For FISH purposes, the biofilm flocks were harvested and fixed as previously described on site with 3% (v/v)% formaldehyde (Rahlff et al. 2021), stored at −20°C, and deposited on a slide for hybridization. For *Ca. A*. crystalense (CG), cells were filtered onto 0.2 μm PTFE filters (Polytetrafluorethylene) and fixed on site with 3% (v/v)% formaldehyde and stored at −80°C as described previously (Schwank et al. 2019). The samples from CG and MSI were taken in August 2021 and February 2022, respectively. For both systems, the 16S rRNA probe ‘SMARCH714’ (Moissl et al. 2003) was labelled with Atto488. The episymbiont *Ca. Huberiarchaeum* crystalense was labelled with a specifically designed Cy3 probe called ‘HUB1206’ (Schwank et al. 2019). The FISH procedure was carried out as previously described by (Schwank et al. 2019). Cells were counterstained with DAPI (4 μg mL^−1^).

Image analysis was carried out with a Zeiss Axio Imager M2m epifluorescence microscope (X‐Cite XYLIS Broad Spectrum LED Illumination System, Excelitas) equipped with an Axio Cam MRm and a Zen 3.4 Pro software (version 3.4.91.00000). Imaging was carried out by using the 100×/1.3 oil objective EC‐Plan NEOFLUAR and three different filter sets: 09 for achieving the 16S rRNA signals of *Ca. Altiarchaea*, 43 Cy3 for the detection of *Ca. Huberiarchaea* signals and 49 DAPI for imaging *Ca. A*. crystalense/hamiconexum cells and *Ca. H*. crystalense cells.

## Author Contributions

**Sarah P. Esser:** conceptualization, investigation, writing – original draft, methodology, validation, visualization, data curation, writing – review and editing. **Victoria Turzynski:** visualization, investigation. **Julia Plewka:** methodology, formal analysis, investigation. **Julia Nuy:** visualization. **Carrie J. Moore:** investigation, methodology, formal analysis. **Indra Banas:** visualization, investigation. **André R. Soares:** investigation, formal analysis. **Janey Lee:** investigation, methodology. **Tanja Woyke:** investigation, methodology. **Alexander J. Probst:** conceptualization, funding acquisition, writing – original draft, methodology, formal analysis, supervision, project administration, writing – review and editing.

## Conflicts of Interest

The authors declare no conflicts of interest.

## Supporting information


**Data S1.** emi470096‐sup‐0001‐Supinfo.

**Table S1.** emi470096‐sup‐0002‐TableS1.

**Table S2.** emi470096‐sup‐0003‐TableS2.

**Table S3.** emi470096‐sup‐0004‐TableS3.

**Table S4.** emi470096‐sup‐0005‐TableS4.

## Data Availability

All metatranscriptomics datasets are published under the accession numbers SAMN14515498, SAMN14515403, SAMN14515402 for CG and under the BioProject number PRJNA1005487 with the individual Accession numbers SRX21390066, SRX21390067, and SRX21390068 for MSI. The accession numbers of metagenomic assembled genomes are listed in Table S1.
