# Supplementary material for: Differential Expression of Core Metabolic Functions in Candidatus Altiarchaeum Inhabiting Distinct Subsurface Ecosystems
Source: Environ Microbiol Rep. 2025 Jun 3;17(3):e70096. doi: 10.1111/1758-2229.70096 (PMC12133352; doi:10.1111/1758-2229.70096)
Supplement: Supplementary file 1 — Data S1. emi470096‐sup‐0001‐Supinfo. [file EMI4-17-e70096-s004.docx]

**Differential expression of core metabolic functions in DPANN Archaea of distinct subsurface ecosystems**

Sarah P. Esser^1,*^, Victoria Turzynski^1^, Julia Plewka^1^, Julia Nuy^1^, Carrie Moore^1^, Indra Banas^1^, André R. Soares^1,4^, Janey Lee^2^, Tanja Woyke^2,3^, Alexander J. Probst^1,4,5,^*

^1^ Environmental Metagenomics, Research Centre One Health Ruhr of the University Alliance Ruhr, Faculty of Chemistry, University Duisburg-Essen, 45141 Essen, Germany

^2^ DOE Joint Genome Institute, Lawrence Berkeley National Laboratory, One Cyclotron Rd, Berkeley, CA, 94720, USA

^3^ University of California Merced, Department of Life and Environmental Sciences, Merced, CA 95343, USA

^4^ Centre of Water and Environmental Research (ZWU), University of Duisburg-Essen, Universitätsstraße 5, 45141 Essen, Germany

^5^ Center for Medical Biotechnology (ZMB), University of Duisburg-Essen, 45141 Essen, Germany

^*^co-corresponding authors: sarah.esser@uni-due.de, alexander.probst@uni-due.de

Content:

1. Supplementary Figures
2. List of supplementary Tables
3. References
4. **Supplementary Figures**


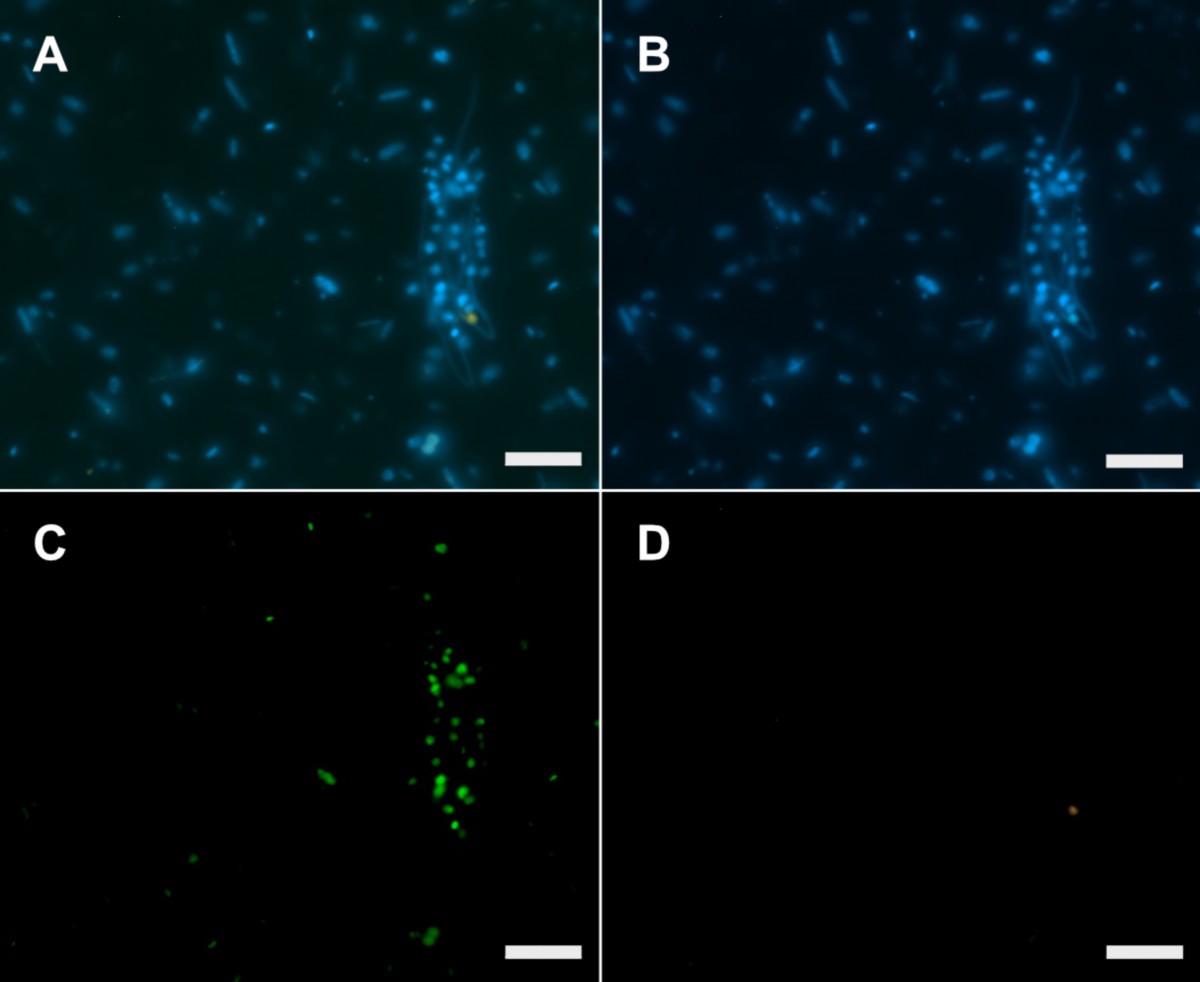


**Figure S1|** Fluorescence *in situ* hybridization images of *Ca.* Altiarchaeum crystalense and *Ca.* Huberiarchaeum crystalense in Crystal Geyser. **A** Merged FISH images as shown in Fig. 1. **B** DAPI staining of the sample. **C** 16S rRNA of *Ca.* Altiarchaeum labeled using the SMARCH714 probe^1^ with Atto488. **D 16S rRNA of** *Ca.* Huberiarchaeum crystalense labeled using the Hub1206 probe^2^ and Cy3. Scale bar = 10 µm.


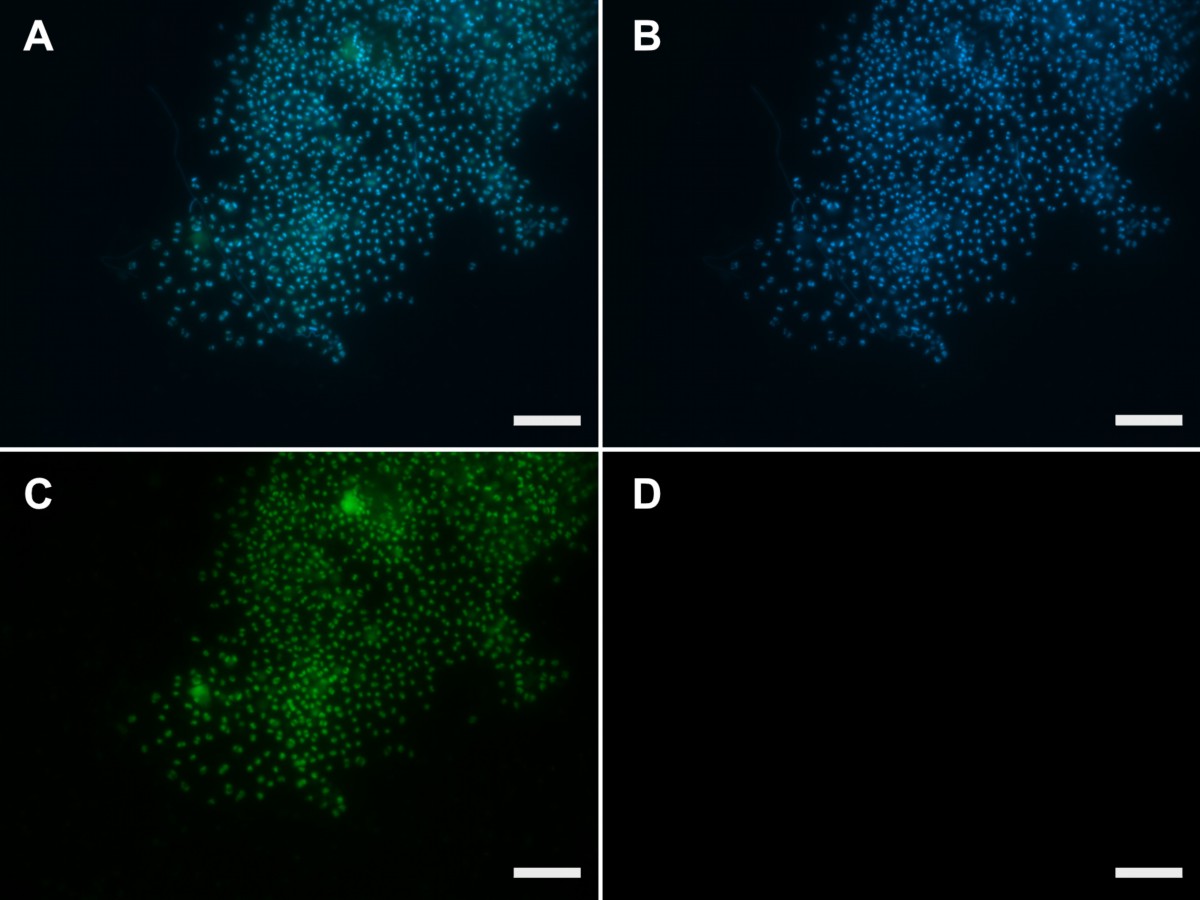


**Figure S2|** Fluorescence *in situ* hybridization images of *Ca.* Altiarchaeum hamiconexum **A** Merged FISH images as shown in Fig. 1. **B** DAPI staining of the sample. **C** 16S rRNA of *Ca.* Altiarchaeum labeled using the SMARCH714 probe^1^ with Atto488. **D 16S rRNA of** *Ca.* Huberiarchaeum crystalense labeled using the Hub1206 probe^2^ and Cy3. Scale bar = 10 µm.

**Figure S3|** Difference in expression profile of ribosomal proteins derived from metatranscriptomics data of CG (blue) and MSI (magenta), respectively. Visualization was performed with ggplot2^3^ in R studio^4,5^.

**Figure S4|** Difference in expression profile of proteins related to cell-defense and derived from metatranscriptomics data of CG (blue) and MSI (magenta), respectively. Visualization was performed with ggplot^3^ in R studio^4,5^.

**Figure S5|** Difference in expression profile of replication, translation and transcription related proteins derived from metatranscriptomics data of CG (blue) and MSI (magenta). Visualization was performed with ggplot^3^ in R studio^4,5^.

**Figure S6|** Difference in expression profile of proteins classified as “domain of unknown function” and derived from metatranscriptomics data of CG (blue) and MSI (magenta). Visualization was performed with ggplot^3^ in R studio^4,5^.

**Figure S7|** Difference in expression profile of energy related proteins derived from metatranscriptomics data of CG (blue) and MSI (magenta). Visualization was performed with ggplot^3^ in R studio^4,5^.

**Figure S8|** Difference in expression profile of carbon, nitrogen and sulfur metabolism related proteins derived from metatranscriptomics data of CG (blue) and MSI (magenta). Visualization was performed with ggplot^3^ in R studio^4,5^.

**Figure S9|** Difference in expression profile of membrane proteins derived from metatranscriptomics data of CG (blue) and MSI (magenta). Visualization was performed with ggplot^3^ in R studio^4,5^.

**Figure S10|** Difference in expression profile of vitamin related proteins derived from metatranscriptomics data of CG (blue) and MSI (magenta). Visualization was performed with ggplot^3^ in R studio^4,5^.

**Figure S11|** Inter and intra ecosystem comparison of the mean normalized gene expression of CG’s and MSI’s shared gene clusters (Kruskal-Wallis Anova, *p*-values within figure). CG showed a significantly higher expression of the core metabolic pathways then MSI. Data visualization was performed with ggplot^3^ in R studio^4,5^.

**Figure S12|** Principal Coordinate Analyses (PCoA) of normalized gene expression within the metatranscriptomic dataset. Compared are different calculations of the dissimilarity matrices depending on the respective model listed in the PCoA titles. All variants of the PCoAs show that the datasets of Crystal Geyser and Muehlbacher sulfidic spring gene clusters separated from each other. Data visualization was performed with ggplot^3^ in R studio^4,5^. Generally, the above 90% of the changes in the datasets can be explained by the environment.

1. **List of supplementary tables**

**Table S1 |** Normalization factors calculated based on ten ribosomal proteins that are shared at 80% amino acid similarity in CG and MSI. The abundance was calculated by mapping the quality filtered raw reads against the respective assembled scaffold carrying encoded ribosomal proteins.

**Table S2 |** List of read and genome accession numbers published^6^ before or with this manuscript. Genomes which are released on Figshare have been newly binned from Probst et al. 2017^7^ for this study.

**Table S3 |** Raw and normalized coverages of shared gene clusters in CG and MSI. Normalization was done based in the in Table S1 calculated normalization factors for 364 shared gene clusters at 80% amino acid similarity. Gene clusters resulting in uncharacterized or proteins of unknown functions have been excluded in the table. The mapping result is based on the representative gene and mapping of raw reads to the representative scaffold.

**Table S4 |** Gene annotations of shared gene clusters in CG and MSI based the FunTaxDB 1.2. [accessed September 2022]^8^.

1. **References**

1. Moissl, C., Rudolph, C., Rachel, R., Koch, M. & Huber, R. In situ growth of the novel SM1 euryarchaeon from a string-of-pearls-like microbial community in its cold biotope, its physical separation and insights into its structure and physiology. *Archives of Microbiology* **180**, 211–217 (2003).

2. Schwank, K. *et al.* An archaeal symbiont-host association from the deep terrestrial subsurface. *The ISME Journal* **13**, 2135–2139 (2019).

3. Wickham, H. *Ggplot2: Elegant Graphics for Data Analysis*. (Springer, 2016).

4. Team, R. C. *R: A Language and Environment for Statistical Computing*. (Vienna, Austria, 2013).

5. Posit team. Rstudio: Integrated Development Environment for R. Posit Software, PBC (2022).

6. Probst, A. J. *et al.* Differential depth distribution of microbial function and putative symbionts through sediment-hosted aquifers in the deep terrestrial subsurface. *Nature Microbiology* **3**, 328–336 (2018).

7. Probst, A. J. *et al.* Genomic resolution of a cold subsurface aquifer community provides metabolic insights for novel microbes adapted to high CO2 concentrations. **19**, 459–474 (2017).

8. Bornemann, T. L. V., Esser, S. P., Stach, T. L., Burg, T. & Probst, A. J. uBin – a manual refining tool for genomes from metagenomes. *Environmental Microbiology* (2023) doi:10.1111/1462-2920.16351.
